# Sample Entropy of Human Gait Center of Pressure Displacement: A Systematic Methodological Analysis

**DOI:** 10.3390/e20080579

**Published:** 2018-08-06

**Authors:** Samira Ahmadi, Nariman Sepehri, Christine Wu, Tony Szturm

**Affiliations:** 1 Department of Mechanical Engineering, University of Manitoba, Winnipeg, MB R3T 5V6, Canada; 2 Department of Physical Therapy, College of Rehabilitation Sciences, University of Manitoba, Winnipeg, MB R3E 0T6, Canada

**Keywords:** sample entropy, treadmill walking, center of pressure displacement, dual-tasking

## Abstract

Sample entropy (SampEn) has been used to quantify the regularity or predictability of human gait signals. There are studies on the appropriate use of this measure for inter-stride spatio-temporal gait variables. However, the sensitivity of this measure to preprocessing of the signal and to variant values of template size (*m*), tolerance size (*r*), and sampling rate has not been studied when applied to “whole” gait signals. Whole gait signals are the entire time series data obtained from force or inertial sensors. This study systematically investigates the sensitivity of SampEn of the center of pressure displacement in the mediolateral direction (ML COP-D) to variant parameter values and two pre-processing methods. These two methods are filtering the high-frequency components and resampling the signals to have the same average number of data points per stride. The discriminatory ability of SampEn is studied by comparing treadmill walk only (WO) to dual-task (DT) condition. The results suggest that SampEn maintains the directional difference between two walking conditions across variant parameter values, showing a significant increase from WO to DT condition, especially when signals are low-pass filtered. Moreover, when gait speed is different between test conditions, signals should be low-pass filtered and resampled to have the same average number of data points per stride.

## 1. Introduction

Entropy measures quantify the regularity or predictability of a time series [1,2,3,4,5]. Larger entropy values indicate less regularity or predictability in a time series. These measures have been used in gait analysis, and have been shown to discriminate between fallers and non-fallers [6], older and younger adults [7,8] and walk only (WO) and dual-task (DT) walking condition [7]. Various entropy measures have been proposed based on Shannon’s entropy [9] and its successor method, Approximate Entropy [10]. One of the most commonly methods used to study gait function is Sample Entropy (SampEn) [3].

Earlier application of SampEn was used to examine various inter-stride spatio-temporal gait variables, derived from endpoints of the gait cycle (heel strikes), for example, stride time and step length signals [11]. It has also been shown that there may be temporal scales in changes that occur in spatio-temporal gait variables [12]. However, these signals lack intra-stride information, which represents important passive and active gait control process [7]. Recent gait studies have examined “whole” gait signals (entire time series as opposed to stride-to-stride gait variables), such as trunk linear acceleration [1,6,13,14], joint angular positions [15] and center of pressure of feet displacement [7,16].

Center of pressure displacement in the mediolateral direction (ML COP-D) has been extensively used to examine the balance performance during standing conditions (base of support stationary) [17,18,19]. However, its usage in gait analysis is limited to a few studies [1,7,16,20]. This might be due to the limitation of the facilities that collect data on a treadmill or during overground walking. ML COP-D is capable of representing both single and double support phases of the gait cycle. It has also been shown that SampEn of ML COP-D better distinguishes between different treadmill walking conditions as compared to the SampEn of other commonly used signals (e.g., trunk linear acceleration) [7].

A few studies have investigated how variant template size (*m*), tolerance size (*r*), and data length (*N*) would affect SampEn of short [11] and long [21] time series when using inter-stride spatio-temporal gait variables. It was shown that SampEn values are dependent on the combination of *m* and *r*, and not on *N* [11,21]. However, no study has investigated the effect of parameter selection on SampEn of the human gait whole signals, such as ML COP-D or trunk linear acceleration, over an appropriate amount of continuous strides. During continuous, steady-state gait, these signals are similar in nature with a few dominant frequencies and have consistent fluctuations from stride to stride. Considering the increasing use of SampEn in analyzing human gait whole signals, it is essential to investigate how parameter selection would affect the outcomes. The importance of this investigation stems from the fact that parameter selection for calculating SampEn of whole gait signals, in many studies, is based on those that have analyzed inter-stride gait variables [8,13].

Most studies which have examined the effect of aging or dual-tasking on gait function, use self-paced walking and do not control for gait speed. Self-paced walking results in different walking speeds and, therefore, each walking condition will have a different average number of data points per stride. It has been shown that gait speed is significantly reduced during dual-task walking compared to walk only trials [22] and it has been reported that speed has a significant effect on measures of dynamical systems, such as the largest Lyapunov exponent [23,24,25]. Moreover, researchers have used different sampling frequencies when collecting target whole signals, which in turn have resulted in a different average number of data points per stride. It is unknown whether a different average number of data points per stride caused by varying walking speed or sampling rate would affect SampEn. Furthermore, many researchers have opted to apply SampEn, or other entropy measures, to raw unfiltered signals [8,16,26] to avoid losing or altering information due to filtering. While others have filtered the high-frequency components of trunk linear acceleration signal using a cut-off frequency of 20 Hz [14,27]. Therefore, investigating the effect of filtering would also be beneficial.

The first objective of this study is to systematically examine the sensitivity of SampEn of ML COP-D signals, obtained during treadmill walking, to variant *m*, *r*, and sampling rate values. The second objective is to determine the effect of the choice of low-pass filtering and data resampling, to have the same average number of data points per stride, on the SampEn of ML COP-D signals. Discriminatory ability of SampEn will be examined through comparing walk only condition to dual-task walking, which has been shown to adversely affect gait performance [28,29].

## 2. Materials and Methods

### 2.1. Experimental Procedure

A convenience sample of 29 healthy young participants (eight females, 28.3 ± 2.7 years, 173.4 ± 8.8 cm, 69.7 ± 14.2 kg, mean ± standard deviation (SD)) was recruited. They were screened to ensure that no participant had any illnesses, neuromuscular injuries or previous surgeries that might affect their balance and gait. The University of Manitoba Human Research Ethics Committee approved the study and all participants signed the informed consent form prior to the tests.

Participants were asked to walk on an instrumented Bertec treadmill (Bertec Corporation, Columbus, OH, USA) under three different walking conditions:

(a) Walk only (WO) trial of 1 min at a speed of 1.0 m/s, and
(b) Dual-task (DT) walking trial of 1 min at a speed of 1.0 m/s, which is described below, and
(c) Walk only trial of 1 min at a speed of 1.3 m/s (WO-1.3).

**Figure 1:**
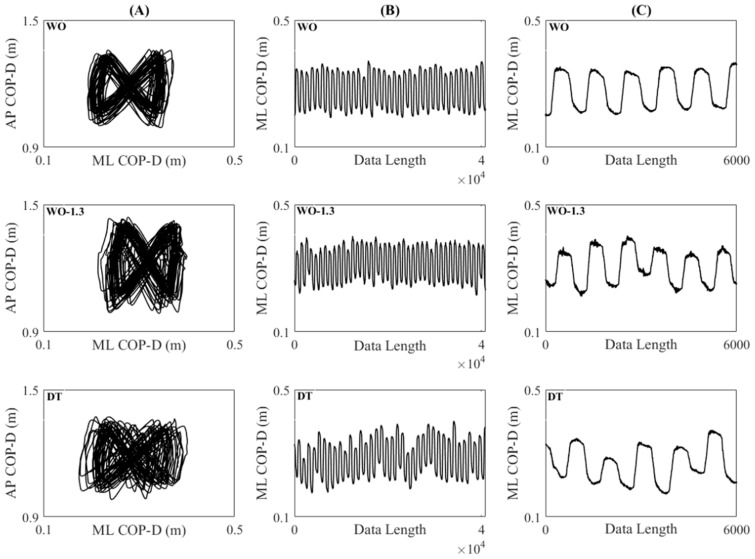
Trajectory of the center of pressure under WO, WO-1.3, and DT conditions: (**A**) The low-pass filtered trajectory of center of pressure displayed as AP COP-D vs. ML COP-D, (**B**) Filtered ML COP-D, (**C**) Several strides of unfiltered ML COP-D.

Center of pressure displacements in the mediolateral (ML COP-D) and anteroposterior (AP COP-D) directions (Figure 1) were calculated from the force and moment components, which were sampled at 1000 Hz. Forty seconds of each signal, which contained at least 30 strides [16], were used after discarding approximately the first 4 strides.

During all walking trials, participants viewed an 80 cm computer monitor positioned 1 meter away at eye level. During the WO trials, participants watched a scenery video to maintain gaze and head position relative to the monitor. For the purpose of hands-free interaction with game activities, a commercial inertial-based wireless mouse (Elite mouse, SMK Electronics, Chula Vista, CA, USA) was mounted on a plastic headband worn by each participant. Therefore, during walking, the head rotation was used to control the motion of the computer cursor. The goal of the game was to move a game paddle horizontally to interact with moving game objects. The game objects were categorized as designated targets or designated distractors, with the shape of a soccer ball and dotted sphere, respectively. They appeared at random locations at the top of the display every 2 s and moved diagonally toward the bottom of the display. In response to each “game event” (target appearance), the participant produced a head rotation (i.e., rotation of the motion-sense mouse) to move the game paddle (left/right) to catch the target objects and avoid the distractors. For a full description of the interactive cognitive computer game, see Szturm et al. [29].

### 2.2. Sample Entropy

SampEn (m, r, N) [3] of a dataset of length N is the negative natural logarithm of the conditional probability of two successive counts of similar pairs (Chebyshev distance less than a tolerance size of *r*) of template size m and m+1 without allowing self-matches. SampEn is calculated as follows [7]; consider a time series of length *N* given below:

(1) u=u(1),u(2),…,u(N) or u={u(j):1≤j≤N}

First, *m* value is chosen to construct series of pairs, size *m* as:

(2) $$X_{m}\left(i\right)=\left\{u\left(i+k\right):0\leq k\leq m-1\right\},\;1\leq i\leq N-m+1$$

Next, matching templates are found by comparing their chebyshev distance to a pre-determined *r* value while excluding self-comparison. Next, a variable called $B_{i}$ is built which is the number of pairs satisfying the aforementioned criteria:

(3) $$B_{i}^{m}\left(r\right)=\frac{1}{N-m-1}\;\left(\#\;\mathrm{of}\;d\left|X_{m}\left(i\right)-X_{m}\left(j\right)\right|\;\leq r,\;\mathrm{where}\;j=1:N-m\;\&\;i\neq j\right)$$

(4) $$d\left|X_{m}\left(i\right)-X_{m}\left(j\right)\right|=\max\left\{\left|u\left(i+k\right)-u\left(j+k\right)\right|:0\leq k\leq m-1\right\}$$

Next, $B^{m}\left(r\right)$ is defined as:

(5) $$B^{m}\left(r\right)=\frac{1}{N-m}\overset{N-m}{\underset{i=1}{\sum }}B_{i}^{m}\left(r\right)$$

This process is repeated for m+1 and *r* to form $A^{m}\left(r\right)$:

(6) $$A_{i}^{m}\left(r\right)=\frac{1}{N-m-1}\;\left(\#\;\mathrm{of}\;d\left|X_{m+1}\left(i\right)-X_{m+1}\left(j\right)\right|\;\leq r,\;\mathrm{where}\;j=1:N-m\;\&\;i\neq j\right)$$

(7) $$A^{m}\left(r\right)=\frac{1}{N-m}\overset{N-m}{\underset{i=1}{\sum }}A_{i}^{m}\left(r\right)$$

Lastly, SampEn is calculated based on $B^{m}\left(r\right)$ and $A^{m}\left(r\right)$ as

(8) $$\mathrm{SampEn}\;\left(m,r,N\right)=\;-\ln\frac{A^{m}\left(r\right)}{B^{m}\left(r\right)}$$

### 2.3. Data Analysis

This study consists of two parts. In the first part, the sensitivity of SampEn to changing *m*, *r*, and sampling rate was investigated when comparing WO to DT. Two methods were used to downsample signals from 1000 Hz to lower sampling rates (Table 1). The goal was to downsample signals by factors of 1, 2, 4, 8, 16 and 32. The first method, decimation (D) by a factor of *f*, used an eighth-order low-pass Chebyshev Type I filter, which filtered the signal in forward and reverse directions to remove phase distortions and then selected every *f*th point (MATLAB command *decimate*). The filter had a normalized cut-off frequency of 0.8/*f*. This method was chosen to avoid aliasing distortion that might occur by simply downsampling a signal.

The second method, filtering-and-downsampling (FD) by a factor of *f*, used a second-order Butterworth low-pass filter with a cut-off frequency of 30 Hz, and then downsampled the signal by a factor of *f* (MATLAB command *downsample*). Butterworth low-pass filter is the most common filter used in the literature to reduce the effect of noise [30] along with maintaining the variability in the lower range frequencies where the musculoskeletal motion occurs [31]. A nonparametric PSD estimator, Welch’s algorithm, was used to obtain the cut-off frequency. The dominant peak was at 0.89 ± 0.06 Hz (mean ± SD) for WO, 0.91 ± 0.06 for DT, and 0.99 ± 0.06 for WO-1.3. The last peak before noise floor occurred in the 8–15 Hz frequency range. Therefore, 15 Hz was considered as the highest frequency component and 30 Hz was used as the cut-off frequency.

The two methods yielded approximately the same results with respect to the low-pass filtering for f=32. Therefore, the first five *f* values could shed light on the effect of low-pass filtering prior to the calculation of SampEn.

SampEn was calculated using all combinations of parameter values, m=2, 4, 6, 8, 10 and *r* = 0.2 and 0.3 × standard deviation (SD) of all the time series, and for all downsampling factors f=1, 2, 4, 8, 16, 32, and for both decimated and filtered-and-downsampled signals of WO and DT condition. The present investigation was based on more *m* and *f* values in the selected ranges. However, the necessity for statistical analysis with the purpose of studying the discriminatory ability of SampEn, led to choosing fewer parameter values (levels within a factor); e.g., five levels versus nine levels for template size (*m* = 2~10). In a previous study [11], m=2, 3, 4 were tested when SampEn was applied to the inter-stride spatio-temporal gait variables. The present work included more *m* values to study the SampEn of the entire gait signals and not just times at heel strike or step distances. It was hypothesized that larger *m* values could better discern changes when there is a much greater number of data points per gait cycle or stride. Additionally, unlike ApEn, SampEn decreases almost monotonically with increasing *r* value [3,11] and 0.1–0.3 times the standard deviation has been suggested for inter-stride spatio-temporal gait variables [11]. The current analysis was based on r=0.1×SD, r=0.2×SD and r=0.3×SD. However, when the parameter value r=0.1×SD was used, many SampEn values converged to infinity. Therefore this level was not included in the results. Large *r* values were not included because they result in much smaller SampEn values for each condition, i.e., more matched templates, which diminish the discriminatory ability of SampEn.

In the second part, the effect of low-pass filtering and resampling, to have the same average number of data points per stride, was investigated. SampEn of ML COP-D signal of WO, DT, and WO-1.3 was calculated using m=4, r=0.2×SD, and f=8 (based on the results of the first part). Four methods of preprocessing were used for each condition;

- decimation (D),
- decimation-and-resampling (D-R),
- filtering-and-downsampling (FD) and,
- filtering-and-downsampling-and-resampling (FD-R).

The average number of data points per stride for WO, DT and WO-1.3 were 142, 140 and 128, respectively. Therefore, 30 strides of each time series were resampled (MATLAB command *resample*) so that all of the signals would have an average of 142 data points per stride.

### 2.4. Statistical Analysis

In the first part of this study, there were 4 factors of within-subject repeated measures, which are 2 levels of walking condition (WO and DT), 2 levels of *r*, 6 levels of *f*, and 5 levels of *m*. The following steps were taken to perform the statistical analysis separately for both decimated and filtered-and-downsampled signals.

- A two-factor repeated measure ANOVA (walking condition*m) was performed at each *f* level while considering the first tolerance level.
- A two-factor repeated measure ANOVA (walking condition*m) was performed at each *f* level while considering the second tolerance level.
- A two-factor repeated measure ANOVA (walking condition*f) was performed at each *m* level while considering the first tolerance level.
- A two-factor repeated measure ANOVA (walking condition*f) was performed at each *m* level while considering the second tolerance level.
- Post hoc pairwise comparisons with Bonferroni correction were performed to examine the effect of dual-tasking at each level.
- Finally, a two-factor (walking condition*r) repeated measure ANOVA was performed at fixed *m* = 4 and *f* = 8 values, which were chosen based on the previous step’s statistical results.

In the second part of this study, two two-factor within-subject ANOVA were used to examine the main and interaction effects of the following factors on SampEn;

- walking condition (WO versus DT) and preprocessing method (D, D-R, FD, FD-R)
- gait speed (1.0 m/s versus 1.3 m/s) and preprocessing method (D, D-R, FD, FD-R)

Normality of all dependent variables was checked using the Shapiro-Wilk normality test. Results confirmed that the data was normally distributed. Statistical analyses were carried out using SPSS software version 24. In all the tests, a *p*-value less than 0.05 was considered significant. A Bonferroni correction was used in the software for multiple comparisons.

## 3. Results

For the vast majority of combinations of parameter values, SampEn of ML COP-D during dual-task walking was significantly larger than that of walk only. In general, SampEn decreased as *m* increased, as *r* increased, and as *f* factor decreased, i.e., as sampling rate increased or as the number of points per stride increased. However, there were a few exceptions, which will be discussed further. The results of the main and interaction effects of walking condition (WO and DT), *m*, and *f* at each *r* value are presented in Table 2 and Table 3. In addition, the results of pairwise comparisons of the significant main effects of walking condition are presented in Table A1 and Table A2. The detailed results for each downsampling method are presented in the following subsections followed by the results of the effects of the preprocessing methods.

### 3.1. Sensitivity of SampEn to Variant Parameter Values When Using Filtering-and-Downsampling

**Figure 2:**
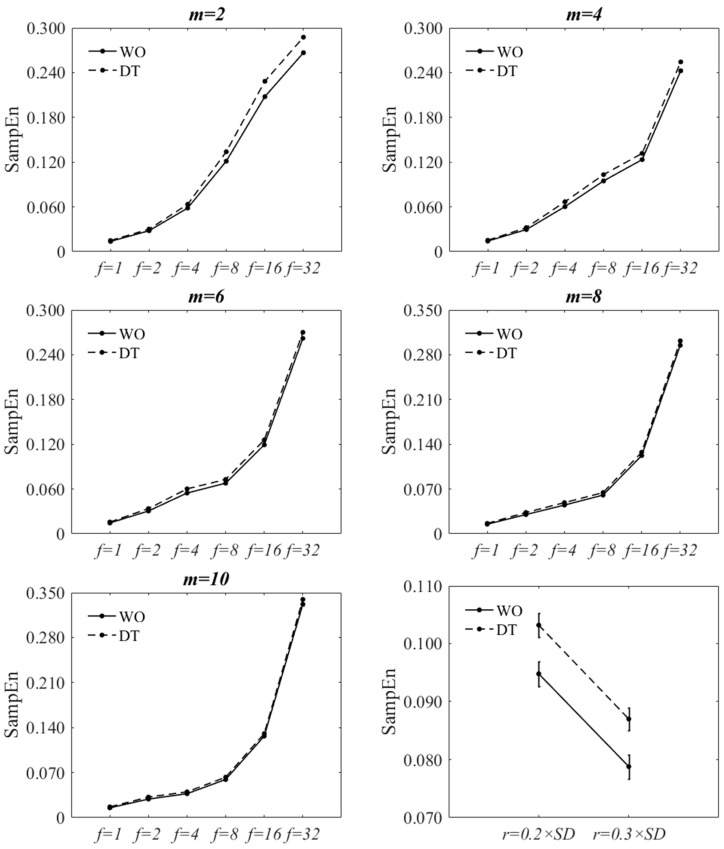
Effect of changing *f* on SampEn of WO and DT at each *m* value at r=0.2×SD. for filtered-and-downsampled ML COP-D. Bottom-right: Effect of *r* on SampEn at f=8 and m=4. The error bars reflect the standard error of the means.

**Figure 3:**
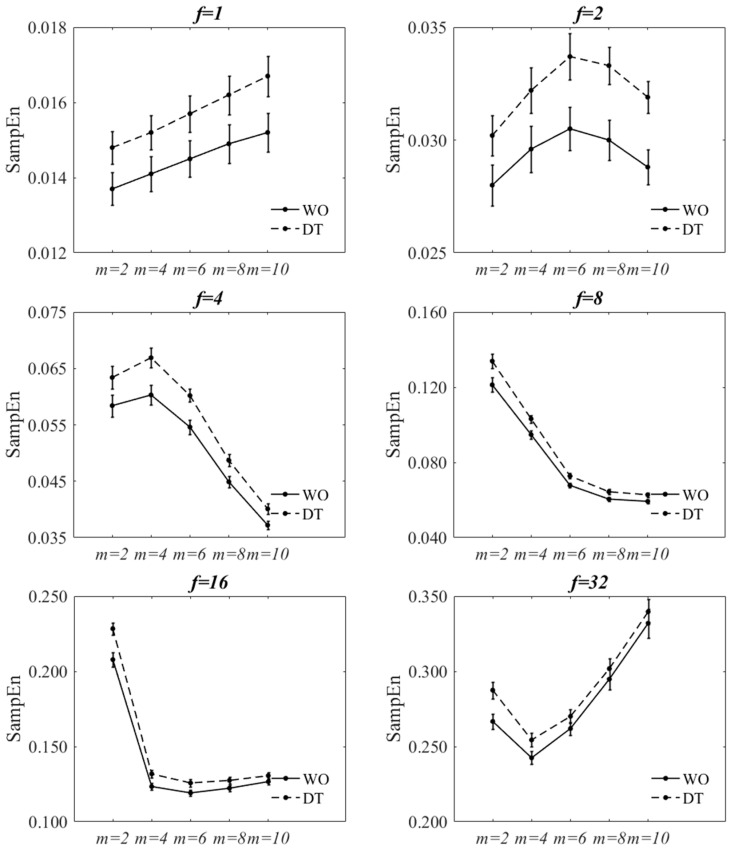
Effect of changing *m* on SampEn of WO and DT at each *f* value at r=0.2×SD for filtered-and-downsampled ML COP-D. The error bars reflect the standard error of the means.

Figure 2 and Figure 3 show the effect of changing *f* and *m* values on SampEn of the filtered-and-downsampled signals at r=0.2×SD. The figures are virtually the same as those of  r=0.3×SD. The statistical results are also reported in Table 2, Table 3, Table A1 and Table A2. A statistically significant interaction was found between walking condition and *f* at m=2 and m=4 for both tolerance values. Since the direction of the changes of SampEn with increasing *f* was increasing for both walking conditions, the interaction effect would signify a difference in the rate of the changes between levels. A statistically significant interaction was found between walking condition and *m* at all *f* values except for; (a) f=4 and f=32 for r=0.2×SD and (b) f=4 and f=8 for r=0.3×SD. At each *f* value, the direction of the changes of SampEn of WO, with respect to *m*, was similar to those of DT. The two-factor repeated measures of ANOVA of walking condition *r showed that there was no significant interaction between the walking condition and the *r* value (*p* = 0.813). However, there was a statistically significant decrease of SampEn with increasing the *r* value (*p* < 0.001). At each *r* value, SampEn of DT was statistically significantly larger than that of WO (*p* < 0.001).

For all *m* values, there was a statistically significant main effect of walking condition and *f* value on SampEn. Similarly, for all *f* values, there was a statistically significant main effect of walking condition and *m* value on SampEn. SampEn significantly increased from WO to DT for most combinations of *r*, *m*, and *f* values except for; (a) 4 out of 30 combinations of *f* and *m* values for r=0.2×SD and (b) 3 out of 30 combinations for r=0.3×SD. Nevertheless, for these exceptions, there was a trend of increased SampEn from WO to DT. Based on the statistical analysis, the increasing effect of dual-tasking on SampEn of ML COP-D signal can be captured for most combinations of *r*, *m*, and *f* values. The only exceptions are m=10 at f=16 and m=6, 8, 10 at f=32.

### 3.2. Sensitivity of SampEn to Variant Parameter Values When Using Decimation

**Figure 4:**
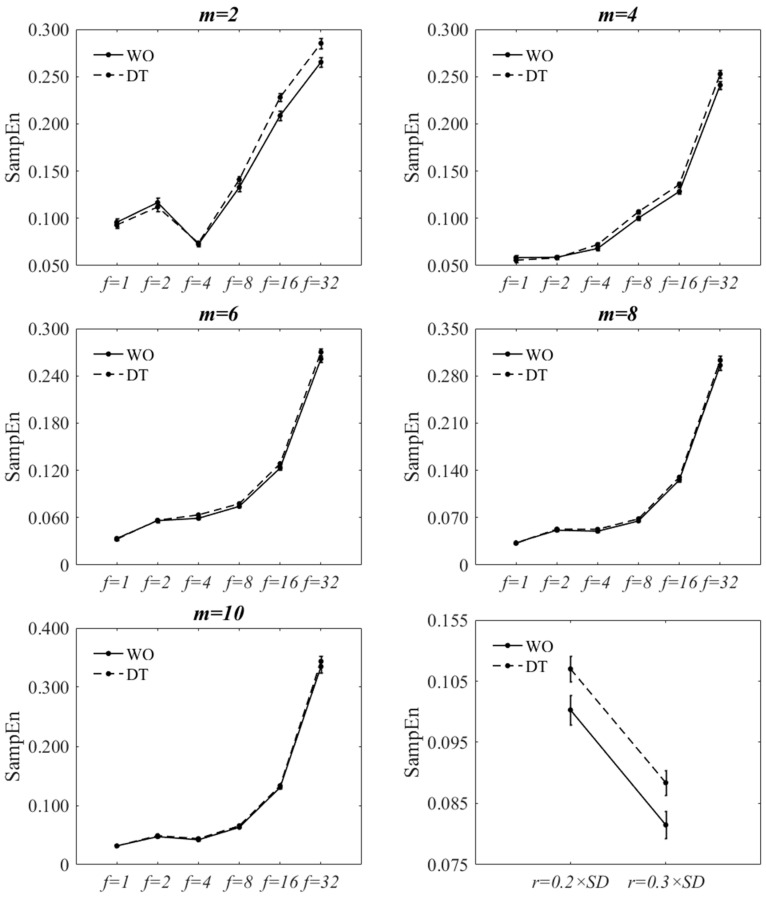
Effect of changing *f* on SampEn of WO and DT at each *m* value at r=0.2×SD for decimated ML COP-D. Bottom-right: Effect of *r* on SampEn at f=8 and m=4. The error bars reflect the standard error of the means.

**Figure 5:**
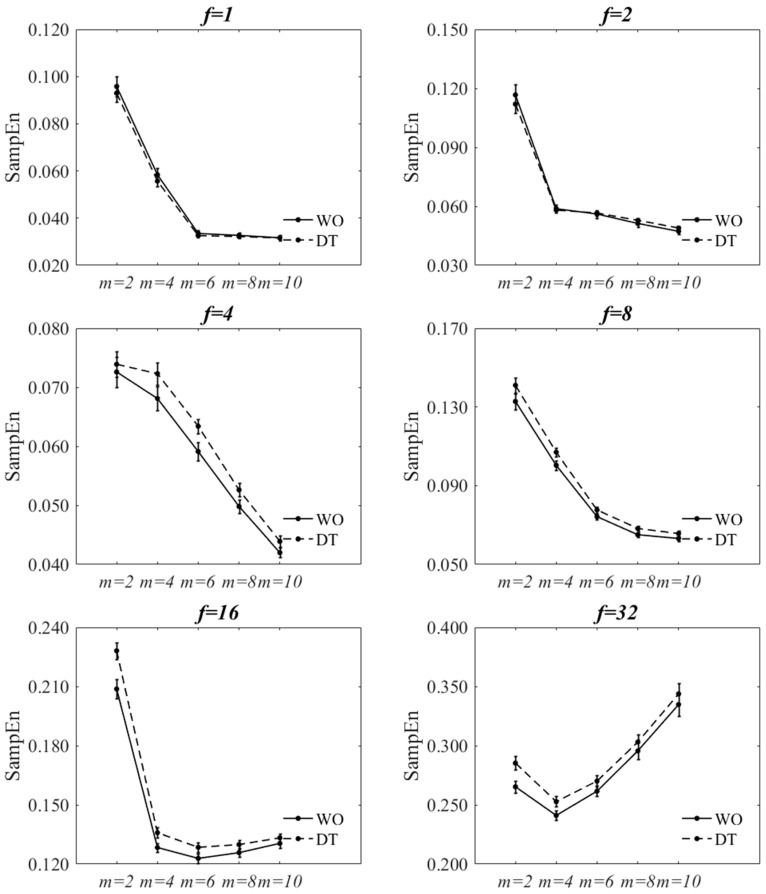
Effect of changing *m* on SampEn of WO and DT at each *f* value at r=0.2×SD for decimated ML COP-D. The error bars reflect the standard error of the means.

Figure 4 and Figure 5 show the effect of changing *f* and *m* values on SampEn of the decimated signal at r=0.2×SD. The figures are virtually the same as those of r=0.3×SD. The statistical results are also reported in Table 2, Table 3, Table A1 and Table A2. A statistically significant interaction was found between walking condition and *f* at m=2, m=4, and m=6 for both tolerance values. The direction of the changes of SampEn with increasing *f* was increasing for both tasks. A statistically significant interaction was found between walking condition and *m* at all *f* values except for; (a) f=4, f=8 and f=32 for r=0.2×SD and (b) f=8 for r=0.3×SD. At each *f* value, the direction of changes of SampEn of WO, with respect to *m*, was the same to those of DT. The two-factor repeated measure of ANOVA of walking condition-*r* showed that there was no significant interaction between the walking condition and the *r* value (*p* = 0.980). However, there was a statistically significant decrease of SampEn with increasing the *r* value (*p* = 0.001). At each *r* value, SampEn of DT was statistically significantly larger than that of WO (*p* < 0.001).

For all *m* values, there was a statistically significant main effect of walking condition (except for m=10) and *f* values on SampEn. And for all *f* values, there was a statistically significant main effect of walking condition (except for f=1 and f=2) and *m* values on SampEn. SampEn significantly increased from WO to DT for most combinations of *m*, *r*, and *f* (4, 8, 16, and 32) values with a few exceptions; smaller *m* values at f=4 and larger *m* values at f=16 and f=32. Nevertheless, SampEn was seen to increase from WO to DT for those exceptions.

### 3.3. Effects of Preprocessing Methods

**Figure 6:**
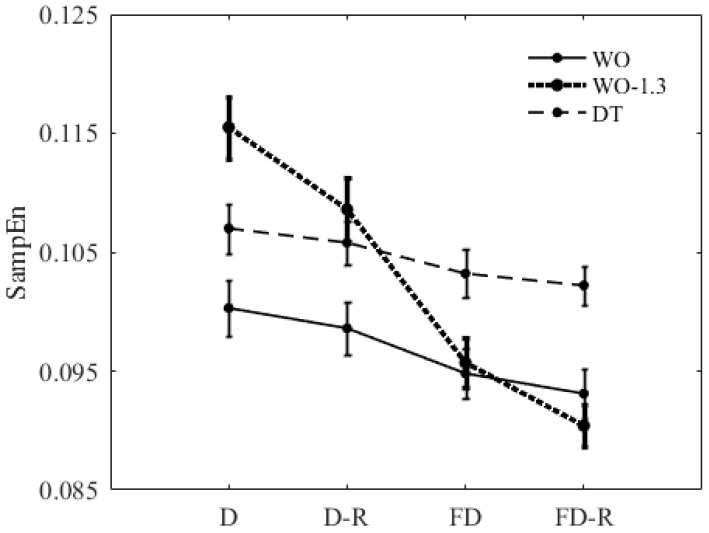
Effects of different preprocessing methods on SampEn of the ML COP-D signal of WO, DT, and WO-1.3 conditions for the combination of f=8, m=4, and r=0.2×SD; The error bars reflect the standard error of the means. D: Decimation; D-R: Decimation-and-Resampling; FD: Filtering-and-Downsampling; and FD-R: Filtering-and-Downsampling-and-Resampling.

The descriptive and statistical results of the walking condition (WO versus DT), walking speed (1.0 m/s versus 1.3 m/s) and preprocessing method (D, D-R, FD, FD-R) on SampEn for the combination of f=8, m=4 and r=0.2×SD are presented in Figure 6 and Table 4.

There was a significant interaction effect of walking speed and method, while no significant interaction of walking condition and method was found. In addition, all three walking condition, speed, and method had a significant main effect on SampEn. Results revealed that resampling signals to have a larger average number of data points per stride had a decreasing effect on SampEn of WO-1.3 signals. However, there was no significant effect of resampling on SampEn of DT and WO signals. The only exception was the significant decrease of SampEn of WO from FD to FD-R. In addition, SampEn significantly decreased by filtering the high-frequency components. Furthermore, SampEn increased significantly from WO to DT when using all four methods. Finally, there was a significant increase in SampEn with increasing walking speed (WO to WO-1.3) only when using decimation or decimation-and-resampling.

## 4. Discussion

The goal of this investigation was to identify the sensitivity of SampEn to variant values of parameters (*m* and *r*, and sampling rate) and two preprocessing methods when applied to ML COP-D signal obtained during treadmill walking under WO and DT conditions. There were three main observations in this study. First, SampEn of ML COP-D consistently increased significantly from WO to DT for most of the combinations of the parameter values and methods of pre-processing. This finding is in agreement with the previous studies [7,16,32]. The only exceptions were m=10 at f=16 and m=6, 8, 10 at f=32 for filtered-and-downsampled ML COP-D. And for decimated ML COP-D, all *m* values at f=1 and f=2, and smaller *m* values at f=4 and larger *m* values at f=16 and f=32. Second, the results demonstrated that walking speed should be controlled when studying the effect of another factor, such as adding a cognitive task. Finally, using a low-pass filter to eliminate high-frequency signal components and smoothing a time series improved the consistency (a significant increase from WO to DT) of SampEn analysis.

The results of this study showed that SampEn of ML COP-D signal increases as participants perform a concurrent cognitive task while walking. Steady-state gait signals are intrinsically periodic with consistent fluctuation from stride to stride. It can be argued that when human gait is disrupted by performing a secondary task or negatively affected by aging or diseases, these fluctuations would change. The unplanned fluctuations might increase during walking under challenging conditions known to cause gait disturbances and stumbles [33]. Another study has reported that SampEn of trunk linear acceleration signal was smaller in older adults who had reported a fall versus non-fallers when tested during overground walking [6]. However, in that study, speed was not controlled among participants or between groups. Reducing one’s walking speed is a consistent strategy to manage threats to balance and when attending to concurrent cognitive tasks. In this regard, a main finding of the present study was that walking speed had a significant effect on SampEn of ML COP-D signal. One reason is an increase in vibrational noise of the treadmill-mounted force plate at higher walking speeds. Increased noise will cause an increase in SampEn. This problem can be solved by preprocessing the signal using a low-pass filter to eliminate high-frequency vibrational noise. Another issue when comparing signals collected at different speeds is that stride time will be reduced as speed increases, and therefore the number of data points per stride will be reduced. The present results of the effect of different *f* values on SampEn demonstrated that SampEn increased as the number of data points per stride decreased. One method to deal with this issue is to resample the signals so that the average number of data points per stride would be the same across different walking speeds. Based on these findings, it is important to control the walking speed when comparing SampEn of gait signal between WO and DT conditions; since people, especially older adults, slow down when they perform a secondary task [34,35].

The results of this study suggest that SampEn benefits from a relative consistency (a significant increase from WO to DT) across different combinations of the variant values of *m*, *r*, and *f*. For chaotic signals like Mackey-Glass system, which resemble periodic time series, entropy values decrease with increasing the *m* values [36]. In the present study, there was a decreasing trend of SampEn of ML COP-D signal with increasing *m*. However, there were exceptions and SampEn values plateaued only at m=4 for f=16. Nevertheless, for most combinations, there was a statistically significant increase of SampEn from WO to DT. With respect to changes in template size at higher sampling rates, SampEn showed an increasing trend with increasing *m*. A possible reason for this behavior may be related to the strong periodicity of the signal. To overcome this, larger template sizes might be chosen for signals collected at higher sampling rates. A similar issue exists for lower sampling rates where SampEn values increased with increasing *m*, but only after a specific *m* value. This suggests that smaller *m* values should be chosen for lower sampling rates. The two tolerance values performed similarly since the smaller one was already larger than the noise level of the signal.

There are three limitations to this study. First, the sample size of the current study (29 participants) which led to performing several 2-factor repeated measure ANOVA instead of for example two 3-factor repeated measure ANOVA. A much larger sample size would be more appropriate to study the effect of four factors each with many levels. Second, this study examined the discriminatory ability of SampEn by only comparing WO to DT conditions. Several other factors, such as aging, should be considered to generalize the results of this study. Finally, only SampEn, which is a single-scale entropy measure, was studied. Multi-scale SampEn analysis [12] or modified SampEn analysis (incorporating a time delay greater than one) [37] would likely yield important findings.

For future studies using the ML COP-D signal, it is recommended to use a low-pass filter prior to the calculation of SampEn. In addition, a sampling rate of 125 Hz or 62 Hz with m=2~6 and r=0.2×SD would be the preferred combinations. For studies testing overground walking where speed is difficult to control, an integer number of strides should be resampled so that the average number of data points per stride remains the same. For research investigations using other gait signals, such as trunk/pelvis linear acceleration, a similar approach should be performed to select the best combinations of *m*, *r*, and *f* values. This study was not designed to investigate the effect of data length on SampEn. Nevertheless, SampEn of whole signals plateaus after a few strides [7] and it has been shown that 30 strides are sufficient to calculate SampEn of whole gait signals [16].

## Figures and Tables

**Table 1:** Summary of downsampling factors (*f*), sampling rates and cut-off frequency for decimation and filtering-and-downsampling methods.

*f*	Sampling Rate (Hz)	Cut-Off Frequency (Hz)	
		Decimation	Filtering-and-Downsampling
1	1000	800	30
2	500	400	30
4	250	200	30
8	125	100	30
16	62	50	30
32	31	25	30

**Table 2:** Main and interaction effects (*p*-values) of “walking condition (W-C)*f” at each *r* and *m* value for FD (Filtered-and-Downsampled) and D (Decimated) ML COP-D. The two conditions are WO and DT. *p*-values in bold indicate a significant difference.

		**W-C**	***f***	W-C*f			**W-C**	***f***	W-C*f
FD r = 0.2×SD	*m* = 2	**<0.001**	**<0.001**	**<0.001**	D r = 0.2×SD	*m* = 2	**0.009**	**<0.001**	**<0.001**
	*m* = 4	**<0.001**	**<0.001**	**0.006**		*m* = 4	**0.009**	**<0.001**	**<0.001**
	*m* = 6	**0.001**	**<0.001**	0.123		*m* = 6	**0.021**	**<0.001**	**0.023**
	*m* = 8	**0.004**	**<0.001**	0.334		*m* = 8	**0.037**	**<0.001**	0.070
	*m* = 10	**0.020**	**<0.001**	0.421		*m* =10	0.075	**<0.001**	0.211
		**W-C**	***f***	W-C*f			**W-C**	***f***	W-C*f
FD r = 0.3×SD	*m* = 2	**<0.001**	**<0.001**	**<0.001**	D r = 0.3×SD	*m* = 2	**0.008**	**<0.001**	**<0.001**
	*m* = 4	**<0.001**	**<0.001**	**<0.001**		*m* = 4	**0.001**	**<0.001**	**<0.001**
	*m* = 6	**0.002**	**<0.001**	0.080		*m* = 6	**0.015**	**<0.001**	**0.034**
	*m* = 8	**0.006**	**<0.001**	0.232		*m* = 8	**0.032**	**<0.001**	0.154
	*m* = 10	**0.020**	**<0.001**	0.387		*m* = 10	0.068	**<0.001**	0.279

**Table 3:** Main and interaction effects (*p*-values) of “walking condition (W-C)*m” at each *r* and *f* value for FD (Filtered-and-Downsampled) and D (Decimated) ML COP-D. The two conditions are WO and DT. *p*-values in bold indicate a significant difference.

		**W-C**	***m***	W-C*m			**W-C**	***m***	W-C*m
FD r = 0.2×SD	*f* = 1	**0.007**	**<0.001**	**0.001**	D r = 0.2×SD	*f* = 1	0.094	**<0.001**	**0.004**
	*f* = 2	**0.002**	**<0.001**	**0.007**		*f* = 2	0.806	**<0.001**	**0.000**
	*f* = 4	**<0.001**	**<0.001**	0.128		*f* = 4	**0.049**	**<0.001**	0.176
	*f* = 8	**<0.001**	**<0.001**	**0.015**		*f* = 8	**0.008**	**<0.001**	0.145
	*f* = 16	**<0.001**	**<0.001**	**0.002**		*f* = 16	**0.001**	**<0.001**	**0.003**
	*f* = 32	**0.007**	**<0.001**	0.070		*f* = 32	**0.005**	**<0.001**	0.114
		**W-C**	***m***	W-C*m			**W-C**	***m***	W-C*m
FD r = 0.3×SD	*f* = 1	**0.011**	**<0.001**	**0.010**	D r = 0.3×SD	*f* = 1	0.329	**<0.001**	**0.044**
	*f* = 2	**0.006**	**<0.001**	**0.000**		*f* = 2	0.938	**<0.001**	**<0.001**
	*f* = 4	**0.001**	**<0.001**	0.170		*f* = 4	**0.049**	**<0.001**	**0.020**
	*f* = 8	**<0.001**	**<0.001**	0.062		*f* = 8	**0.006**	**<0.001**	0.178
	*f* = 16	**<0.001**	**<0.001**	**0.002**		*f* = 16	**0.001**	**<0.001**	**0.004**
	*f* = 32	**0.005**	**<0.001**	**0.006**		*f* = 32	**0.005**	**<0.001**	**0.010**

**Table 4:** Statistical results of the walking condition (WO and DT), walking speed (1.0 m/s and 1.3 m/s) and preprocessing method (D, D-R, FD, and FD-R) on SampEn of ML COP-D for the combination of f=8, m=4, and r=0.2×SD; The top section presents main and interaction effects. The middle section presents the pairwise comparisons between preprocessing methods for each walking condition (WO, WO-1.3, and DT). The bottom section presents the pairwise comparisons between walking conditions for each preprocessing method. *p*-values in bold indicate a significant difference. D: Decimation; D-R: Decimation-and-Resampling; FD: Filtering-and-Downsampling; and FD-R: Filtering-and-Downsampling-and-Resampling.

**Main and Interaction Effects (*p*-Value)**			
	Condition/Speed	Method	Interaction
WO vs. WO-1.3	**0.017**	**<0.001**	**<0.001**
WO vs. DT	**0.002**	**<0.001**	0.057
**Pairwise Comparisons (*p*-Value)**			
	D vs. D-R	D vs. FD	FD vs. FD-R
WO	0.104	**<0.001**	**0.042**
WO-1.3	**<0.001**	**<0.001**	**<0.001**
DT	0.981	**<0.001**	1.000
Method	WO vs. WO-1.3	WO vs. DT	
D	**<0.001**	**0.013**	
D-R	**0.001**	**0.006**	
FD	0.701	**0.001**	
FD-R	0.225	**<0.001**	

## Appendix A

**Table A1:** Descriptive results (mean ± SD) and pairwise comparisons of “walking condition**m*” at r=0.2×SD and each *f* value for FD (Filtered-and-Downsampled) and D (Decimated) ML COP-D. The two conditions are WO and DT. *p*-values in bold indicate a significant difference.

r=0.2×SD		FD			D		
		WO	DT	*p*-Value	WO	DT	*p*-Value
*f* = 1	*m* = 2	0.014 ± 0.002	0.015 ± 0.002	**0.008**	0.096 ± 0.023	0.093 ± 0.020	-
	*m* = 4	0.014 ± 0.002	0.015 ± 0.002	**0.008**	0.058 ± 0.015	0.056 ± 0.012	
	*m* = 6	0.015 ± 0.003	0.016 ± 0.003	**0.008**	0.034 ± 0.007	0.033 ± 0.005	
	*m* = 8	0.015 ± 0.003	0.016 ± 0.003	**0.008**	0.033 ± 0.007	0.032 ± 0.005	
	*m* = 10	0.015 ± 0.003	0.017 ± 0.003	**0.004**	0.032 ± 0.007	0.032 ± 0.004	
*f* = 2	*m* = 2	0.028 ± 0.005	0.030 ± 0.005	**0.008**	0.117 ± 0.029	0.112 ± 0.024	-
	*m* = 4	0.030 ± 0.006	0.032 ± 0.005	**0.008**	0.059 ± 0.011	0.058 ± 0.008	
	*m* = 6	0.031 ± 0.005	0.034 ± 0.006	**0.002**	0.056 ± 0.011	0.057 ± 0.008	
	*m* = 8	0.030 ± 0.005	0.033 ± 0.004	**0.001**	0.051 ± 0.009	0.053 ± 0.006	
	*m* = 10	0.029 ± 0.004	0.032 ± 0.004	**<0.001**	0.047 ± 0.008	0.049 ± 0.005	
*f* = 4	*m* = 2	0.058 ± 0.011	0.063 ± 0.011	**0.008**	0.073 ± 0.014	0.074 ± 0.012	0.568
	*m* = 4	0.060 ± 0.010	0.067 ± 0.009	**0.001**	0.068 ± 0.011	0.072 ± 0.010	**0.042**
	*m* = 6	0.055 ± 0.007	0.060 ± 0.006	**<0.001**	0.059 ± 0.008	0.063 ± 0.007	**0.011**
	*m* = 8	0.045 ± 0.006	0.049 ± 0.006	**0.002**	0.050 ± 0.006	0.053 ± 0.006	**0.035**
	*m* = 10	0.037 ± 0.004	0.040 ± 0.005	**0.002**	0.042 ± 0.004	0.044 ± 0.005	0.058
*f* = 8	*m* = 2	0.121 ± 0.020	0.134 ± 0.021	**0.002**	0.133 ± 0.022	0.141 ± 0.021	**0.042**
	*m* = 4	0.095 ± 0.012	0.103 ± 0.011	**0.001**	0.100 ± 0.013	0.107 ± 0.011	**0.013**
	*m* = 6	0.068 ± 0.007	0.073 ± 0.008	**0.002**	0.074 ± 0.007	0.078 ± 0.009	**0.041**
	*m* = 8	0.061 ± 0.006	0.064 ± 0.007	**0.001**	0.065 ± 0.006	0.068 ± 0.007	**0.008**
	*m* = 10	0.059 ± 0.006	0.063 ± 0.007	**0.001**	0.063 ± 0.007	0.066 ± 0.007	**0.015**
*f* = 16	*m* = 2	0.208 ± 0.025	0.228 ± 0.022	**<0.001**	0.209 ± 0.026	0.228 ± 0.022	**0.001**
	*m* = 4	0.123 ± 0.012	0.132 ± 0.014	**0.001**	0.128 ± 0.012	0.136 ± 0.015	**0.002**
	*m* = 6	0.119 ± 0.012	0.126 ± 0.013	**0.001**	0.123 ± 0.012	0.128 ± 0.013	**0.007**
	*m* = 8	0.122 ± 0.011	0.128 ± 0.012	**0.010**	0.126 ± 0.012	0.130 ± 0.012	**0.049**
	*m* = 10	0.127 ± 0.011	0.131 ± 0.012	0.052	0.130 ± 0.012	0.133 ± 0.012	0.181
*f* = 32	*m* = 2	0.267 ± 0.027	0.288 ± 0.030	**<0.001**	0.265 ± 0.026	0.285 ± 0.030	**0.001**
	*m* = 4	0.243 ± 0.023	0.254 ± 0.024	**0.004**	0.241 ± 0.023	0.253 ± 0.023	**0.004**
	*m* = 6	0.262 ± 0.023	0.270 ± 0.025	0.051	0.262 ± 0.023	0.270 ± 0.025	**0.037**
	*m* = 8	0.295 ± 0.037	0.302 ± 0.036	0.138	0.296 ± 0.037	0.303 ± 0.036	0.105
	*m* = 10	0.332 ± 0.052	0.340 ± 0.045	0.224	0.335 ± 0.053	0.344 ± 0.047	0.154

**Table A2:** Descriptive results (mean ± SD) and pairwise comparisons of “walking condition**m*” at r = 0.3×SD and each *f* value for FD (Filtered-and-Downsampled) and D (Decimated) ML COP-D. The two conditions are WO and DT. *p*-values in bold indicate a significant difference.

r = 0.3×SD		FD			D		
		WO	DT	*p*-Value	WO	DT	*p*-Value
*f* = 1	*m* = 2	0.009 ± 0.002	0.010 ± 0.002	**0.012**	0.058 ± 0.014	0.057 ± 0.011	-
	*m* = 4	0.009 ± 0.002	0.010 ± 0.002	**0.012**	0.034 ± 0.008	0.032 ± 0.006	
	*m* = 6	0.010 ± 0.002	0.010 ± 0.002	**0.011**	0.019 ± 0.003	0.019 ± 0.002	
	*m* = 8	0.010 ± 0.002	0.011 ± 0.002	**0.011**	0.018 ± 0.003	0.018 ± 0.002	
	*m* = 10	0.010 ± 0.002	0.011 ± 0.002	**0.011**	0.018 ± 0.003	0.018 ± 0.002	
*f* = 2	*m* = 2	0.019 ± 0.003	0.020 ± 0.003	**0.012**	0.069 ± 0.016	0.067 ± 0.013	-
	*m* = 4	0.019 ± 0.003	0.021 ± 0.003	**0.011**	0.034 ± 0.006	0.034 ± 0.004	
	*m* = 6	0.020 ± 0.004	0.022 ± 0.004	**0.011**	0.032 ± 0.006	0.032 ± 0.004	
	*m* = 8	0.021 ± 0.004	0.023 ± 0.004	**0.005**	0.031 ± 0.006	0.032 ± 0.004	
	*m* = 10	0.021 ± 0.003	0.023 ± 0.003	**0.002**	0.030 ± 0.005	0.031 ± 0.004	
*f* = 4	*m* = 2	0.038 ± 0.007	0.041 ± 0.007	**0.011**	0.047 ± 0.009	0.048 ± 0.007	0.578
	*m* = 4	0.041 ± 0.008	0.045 ± 0.008	**0.006**	0.046 ± 0.009	0.048 ± 0.008	0.130
	*m* = 6	0.041 ± 0.006	0.045 ± 0.006	**0.001**	0.044 ± 0.007	0.047 ± 0.007	**0.018**
	*m* = 8	0.039 ± 0.005	0.043 ± 0.005	**0.001**	0.041 ± 0.006	0.044 ± 0.005	**0.007**
	*m* = 10	0.035 ± 0.004	0.038 ± 0.004	**0.001**	0.037 ± 0.005	0.040 ± 0.004	**0.008**
*f* = 8	*m* = 2	0.081 ± 0.015	0.088 ± 0.015	**0.008**	0.089 ± 0.017	0.093 ± 0.016	0.127
	*m* = 4	0.079 ± 0.011	0.087 ± 0.010	**0.001**	0.081 ± 0.012	0.088 ± 0.011	**0.005**
	*m* = 6	0.065 ± 0.007	0.070 ± 0.007	**0.001**	0.068 ± 0.008	0.072 ± 0.007	**0.010**
	*m* = 8	0.054 ± 0.004	0.058 ± 0.005	**<0.001**	0.057 ± 0.005	0.060 ± 0.006	**0.003**
	*m* = 10	0.050 ± 0.004	0.053 ± 0.005	**<0.001**	0.053 ± 0.004	0.055 ± 0.005	**0.002**
*f* = 16	*m* = 2	0.161 ± 0.024	0.178 ± 0.024	**0.001**	0.163 ± 0.025	0.178 ± 0.024	**0.002**
	*m* = 4	0.112 ± 0.010	0.120 ± 0.011	**<0.001**	0.115 ± 0.010	0.122 ± 0.012	**0.001**
	*m* = 6	0.100 ± 0.008	0.105 ± 0.010	**<0.001**	0.102 ± 0.009	0.107 ± 0.010	**0.002**
	*m* = 8	0.102 ± 0.009	0.106 ± 0.010	**0.004**	0.104 ± 0.010	0.108 ± 0.011	**0.014**
	*m* = 10	0.106 ± 0.010	0.109 ± 0.010	**0.042**	0.108 ± 0.010	0.111 ± 0.011	0.102
*f* = 32	*m* = 2	0.247 ± 0.024	0.268 ± 0.024	**<0.001**	0.246 ± 0.024	0.265 ± 0.024	**<0.001**
	*m* = 4	0.202 ± 0.018	0.212 ± 0.021	**0.001**	0.201 ± 0.018	0.211 ± 0.020	**0.001**
	*m* = 6	0.219 ± 0.021	0.225 ± 0.022	0.069	0.218 ± 0.021	0.224 ± 0.022	0.066
	*m* = 8	0.240 ± 0.029	0.245 ± 0.030	0.143	0.241 ± 0.030	0.246 ± 0.030	0.156
	*m* = 10	0.255 ± 0.035	0.260 ± 0.034	0.220	0.257 ± 0.036	0.263 ± 0.035	0.211
